# Supplementary material for: Copula-based risk aggregation with trapped ion quantum computers
Source: Sci Rep. 2023 Oct 28;13:18511. doi: 10.1038/s41598-023-44151-1 (PMC10613293; doi:10.1038/s41598-023-44151-1)
Supplement: Supplementary file 2 — Supplementary Information 2. [file 41598_2023_44151_MOESM2_ESM.pdf]

Supplementary Materials

1 Out-of-sample Test in Return Space

In the main text, we presented the results of out-of-sample tests in copula space. The test is more representative of the quantum formula's accuracy because the model is trained in copula space. For the sake of completeness, we also perform the out-of-sample tests in the return space. The results are shown in Fig. 1.

Due to the inverse integral transformation which is nonlinear in nature, and neither convex nor concave, the error bars are amplified, compared with that in the copula space. The inverse transformation also amplifies the error at the tail portion of the distribution. Given the overlapping of confidence intervals and large variability, we can only state that the quantum models in experiment are comparable or relatively better than simulation. However, after the transformation and calculation of severity, it is not easy to identify if the relatively better performance is inherent to the experiment or the effect of the transformation. This is a good question for a more theoretical paper on risk management.

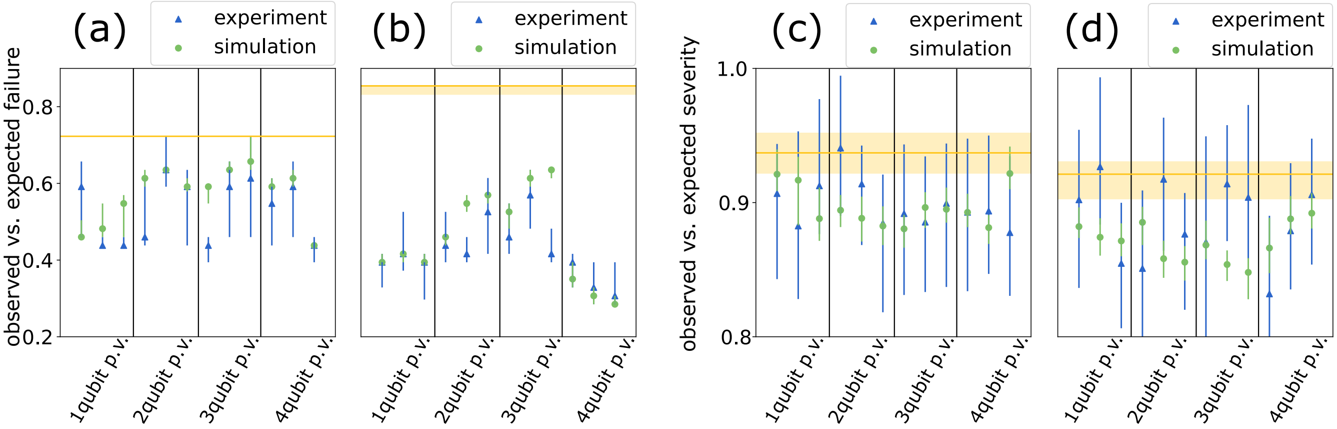

Figure 1. Out of sample test results for different QCBM models in the return space, with different hyper-parameters. See figure 6 in the main text for details of the out-of-sample test. (a) Ratio between observed and expected failures of the portfolio, aggregated with the 3-variable quantum model. (b) Ratio between observed and expected failures of the portfolio, aggregated with the 4-variable quantum model.(c) Ratio between observed and expected severity of the portfolio, aggregated with the 3-variable quantum model. (d) Ratio between observed and expected severity of the portfolio, aggregated with the 4-variable quantum model. The yellow line corresponds to the ratio obtained by the classical copula model. The lighter-yellow-colored region corresponds to the estimation error of the classical model results. Note that both the classical and the simulation approach estimate VaR and ES by 100K generated trials, while the experiment approach estimates VaR and ES by 5K generated trials.

2 Simultaneous Perturbation Stochastic Approximation (SPSA)

We present a brief overview of the SPSA optimizer here for the completeness. A detailed explanation can be found in reference[1].

The SPSA effectively uses gradient descent to optimize systems with many unknown parameters. But unlike the vanilla version of gradient descent that probes partial derivatives with respect to all the parameters, SPSA stochastically samples gradient along a single randomly selected direction using parameter-shift and adjusts the parameters accordingly in each step. Because of the necessity of using parameter-shift rule for the estimation of derivatives in almost all hybrid quantum optimization procedures, as well as inevitable noise from sampling and hardware imperfection, such a stochastic approach provides great efficiency without compromising accuracy.

Specifically, at each optimization step, the gradient $\hat{g}_{\hat{\Delta}}(\hat{\theta})$ along a random direction $\hat{\Delta} = (\delta_1, \ldots, \delta_n)$ is obtained as $\hat{g}_{\hat{\Delta}i}(\hat{\theta}) = \frac{f(\hat{\theta}+c\hat{\Delta})-f(\hat{\theta}-c\hat{\Delta})}{2c\delta_i}$. The optimizer than steps the parameter $\hat{\theta}$ along the direction of the probed stochastic gradient according to $\hat{\theta} = \hat{\theta} + a\hat{g}$. Here δ_i are randomly drawn from ± 1, both a and c are hyperparameters that gradually decrease as the optimization proceeds.

In this work, we follow the recommendation of reference[1] to set both $a = c = 0.1$ before hyperparameter tuning. After tuning, we found that setting $a = c = 0.3$ yields optimal performance, largely due to various sources of noise.

3 Workflow

In this section, we include a flowchart (Fig. 2) that illustrates how the functional modules within our copula framework depend on each other, as well as the data flow. Note within our framework, all data flow are classical.

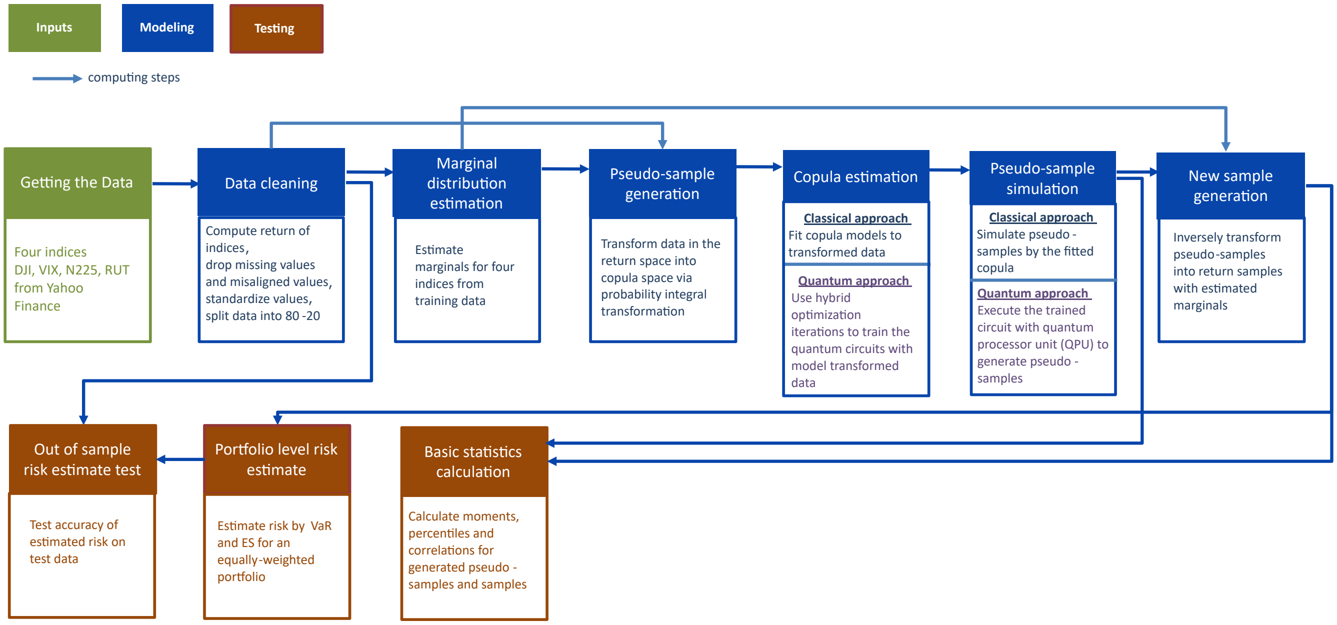

Figure 2. The flowchart illustrates the implementation of the end-to-end test for copula modeling, either with quantum or classical approach. The arrows corresponds to the direction of the data flow.

References

1. Spall, J. C. An overview of the simultaneous perturbation method for efficient optimization. *Johns Hopkins apl technical digest* **19**, 482–492 (1998).
